# Controlled Emissivity Coatings to Delay Ignition of Polyethylene

**DOI:** 10.3390/ma8105349

**Published:** 2015-10-12

**Authors:** Rodolphe Sonnier, Laurent Ferry, Benjamin Gallard, Abderrahim Boudenne, François Lavaud

**Affiliations:** 1 C2MA—Ecole des Mines d’Alès, 6 avenue de Clavières, Alès 30100, France; laurent.ferry@mines-ales.fr (L.F.); Benjamin.gallard@mines-ales.fr (B.G.); 2 CERTES, Université Paris-Est, Créteil Val de Marne, 61 Avenue du Général de Gaulle, 94010 Créteil cedex, France; boudenne@u-pec.fr; 3 Toyal Europe S.A.S.U., Route de Lescun, 64490 Accous, France; francois.lavaud@toyal-europe.com

**Keywords:** thermal-radiative properties, coating, ignition, fire protection

## Abstract

Semi-opaque to opaque films containing small amounts of various aluminium particles to decrease emissivity were easily prepared and coated onto low-density polyethylene (LDPE) sheets. The thermal-radiative properties (reflectivity, transmissivity and absorptivity) of the films were measured and related to the aluminum particles’ content, size and nature. Time-to-ignition of samples was assessed using a cone calorimeter at different heat flux values (35, 50 and 75 kW/m^2^). The coatings allowed significant ignition delay and, in some cases, changed the material behaviour from thermally thin to thick behaviour. These effects are related both to their emissivity and transmissivity. A lower emissivity, which decreases during the degradation, and a lower transmissivity are the key points to ensure an optimal reaction-to-fire.

## 1. Introduction

Among the flammability properties, the ability to ignite is one of the most important to control. Delaying ignition gives people more time to escape from a building or a vehicle during a fire.

The ignition of polymeric materials depends on a complex combination of phenomena controlled by a set of various properties. The ignition occurs when fuel concentration and temperature reach a critical value. Lyon and Quintiere have listed various criteria to assess the time-to-ignition (TTI) of polymers, namely surface temperature, mass loss rate and virtual heat release rate [1]. In the simple geometry of cone calorimeter tests, equations to predict time-to-ignition were proposed for both thermally thick and thin materials [2]. Surface temperature at ignition (which depends on the chemical structure of the material), specific heat, density, thermal conductivity or thickness, emissivity, absorption in-depth [3,4] are material parameters influencing the time-to-ignition. Other phenomena can affect the TTI, like bubbling [5], endothermic decomposition of hydrated fillers and water release and convection in condensed phase.

Fillers also influence TTI by catalyzing degradation [6] or by modifying the thermal-radiative and thermo-physical properties. Fillers can delay ignition by increasing thermal conductivity [7] or accelerate it by decreasing the heat transmitted through the material [8]. In the case of carbon nanotubes, the predominant effect depends on the nanotubes content [7]. In polypropylene filled with various carbon nanoparticles, Dittrich *et al.* have shown that the heat absorption coefficient increases with nanoparticles leading to lower TTI, even if this effect is partly counterbalanced by the increase in thermal conductivity [9,10]. The dispersion of fillers, particularly nanoparticles, also significantly affects the absorption in-depth [9,11].

In aeronautics some metallic particles are well-known to reduce the emissivity of materials preventing a excessively high heating [12,13,14]. Micronized aluminum and copper particles were used to decrease the emissivity of different polymers. The size and shape of these particles influence their efficiency in decreasing the emissivity [12,13]. Another property, called leafing, also has a huge effect. The leafing property corresponds to particles exhibiting a tendency to align themselves parallel to the surface [13].

A few years ago, Schartel *et al.* [15] developed a three-layer coating acting as an infrared mirror (*i.e.*, exhibiting a high reflectivity or a low emissivity). The infrared-mirror layer prepared by physical vapor deposition is very thin (<1 μm) but reduces the heat absorption by up to an order of magnitude. A large increase in TTI was observed for various polymer substrates. Försth *et al.,* have developed a thin coating based on indium tin oxide. This coating reduces the absorptivity of PMMA from 0.93–0.96 to 0.58–0.73 depending to the heat source and the type of PMMA [16]. The TTI in a cone calorimeter test at 25 kW/m^2^ is significantly increased.

In the present work, another strategy is proposed to delay ignition of materials. Thicker films (65–100 μm) of low density polyethylene (LDPE) containing small amounts of aluminum particles are deposited onto an LDPE 4 mm-thick sheet surface. The thermal-radiative properties of the films are measured and related the ignitability of coated LDPE sheets using a cone calorimeter.

## 2. Experimental

### 2.1. Materials

Low density polyethylene is Riblene FL20 from Polimeri Europa.

Aluminum particles are kindly provided by Toyal Europe S.A.S.U. They are embedded into a polyethylene matrix to facilitate processing. Table 1 lists the main data concerning the three grades used (grades named A20, A40 and A60, for which the respective tradenames are Metax NEO NME 020, NME 040 and NME 060).

**Table 1:** Grades used in this study. LDPE: low density polyethylene.

Grades	Coating Polymer	Aluminum Content (wt %)	Particles Medium Size (μm)	Particles Shape
A20	LDPE	70	20	Silver dollar, leafing
A40	LDPE	70	40	Corn flake, non leafing
A60	LDPE	70	60	Corn flake, non leafing

### 2.2. Sample Preparation

The compounds were prepared using a Clextral BC21 co-rotating twin screw extruder (length 1200 mm, length to diameter ratio *L*/*D* = 48). The temperature was increased from the first barrel to the die (from 100 to 230 °C). Screw speed and feed rate were fixed at 150 rpm and 2 kg/h respectively. Once extrusion conditions stabilized, extruded strands were pelletized.

The films were prepared using a Thermofisher Polylab OS Rheomex 19/25 (Thermofisher, Waltham, MA, USA), single mono screw cast extruder. The length of the film die was 270 mm and the temperature was increased from the first barrel to the die (from 160 to 200 °C). The screw speed was 120 rpm. The film was collected onto a chill roll. The appropriate film thickness (65 or 100 μm) was obtained by adjusting the die thickness and the chill roll speed.

The films prepared are listed in Table 2.

LDPE sheets (100 × 100 × 4 mm^3^) were coated with the films using a thermo-compression press. The films were pressed at 180 °C and 100 bars during 10 min.

**Figure 1:**
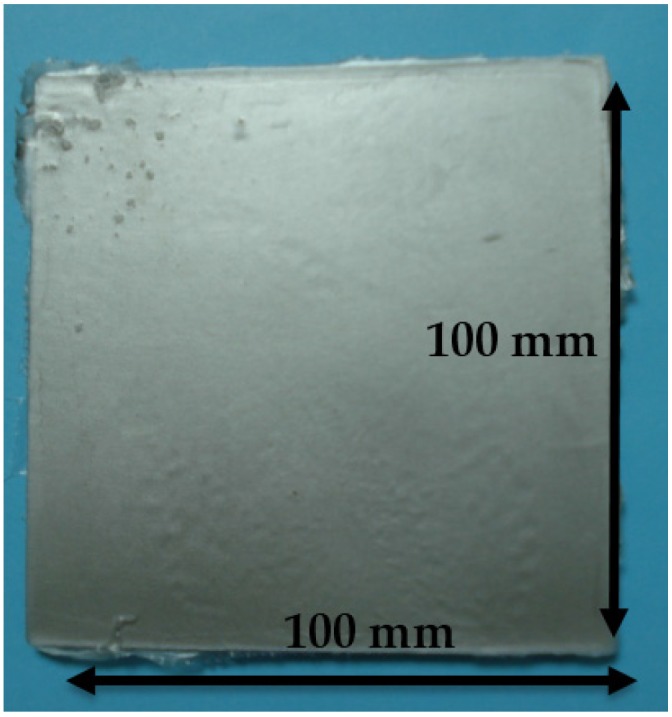
Aspect of the low density polyethylene (LDPE) sheet coated by a thin coating containing aluminum particles.

Figure 1 shows an example of an LDPE sheet coated with a film containing aluminum particles. Even though the surface aspect is not perfect, the film covers the whole surface of the sheet and significantly changes the thermal-radiative properties of the material.

**Table 2:** Films prepared in this study.

Formulations	Aluminum Particles	Grade Content (wt %)	Film Thickness (μm)
1A20-65	A20	1	65
1A20-100	A20	1	100
3A20-100	A20	3	100
5A20-100	A20	5	100
5A40-100	A40	5	100
5A60-100	A60	5	100

### 2.3. Characterization Methods

**Figure 2:**
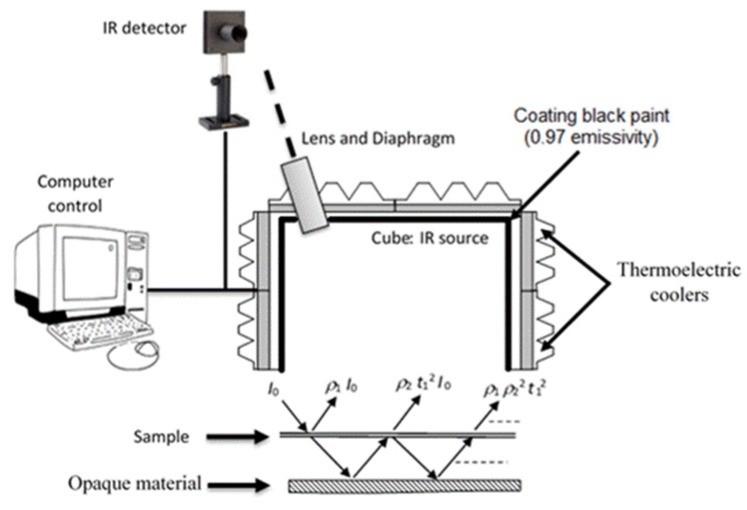
Experimental setup with measurement configuration. IR: infrared.

To measure the thermal-radiative properties of the films, the experimental device used is based on the indirect measurement method, *i.e.*, based on Kirchhoff’s law where the reflectivity is measured and then the emissivity can be deduced. This setup (Figure 2) was developed with collaboration of two laboratories in France [17]. The investigated material is submitted to an isotropic modulated infrared radiation from a Duralumin cube of inner dimensions (10 × 10 × 10) cm^3^, where the internal faces are coated with a black paint of 0.97 emissivity. The infrared source temperature is modulated around 2 °K of the room temperature using four thermoelectric coolers that are series-connected on each face of the cube. The default value of the modulating signal frequency was fixed at 12.5 mHz. The modulated infrared flux emitted by the source and reflected by the upper surface of the studied material was collected through a 1 cm diameter hole drilled in the top plate of the cube. Thus, the reflected intensity is measured by a detector operating in the spectral range 1–40 μm. The large spectral range used allows the measurement of the total hemispherical emissivity, defined in this case as the emissivity of the investigated material.

The measurement protocol begins with a calibration step carried out using a reference material of known emissivity ε_ref_. $\left|{\tilde{U}}_{\text{ref}}\right|$ and $\left|{\tilde{\text{Φ}}}_{\text{s}}\right|$ respectively denote the voltage amplitude (V) and the emitted flux at the modulating frequency (W/m^2^). Therefore:

(1) $$\left|{\tilde{U}}_{\text{ref}}\right|=C\;\left(1-\varepsilon_{\text{ref}}\right)\;{\tilde{\text{Φ}}}_{\text{s}}$$

By application of the Stefan-Boltzmann law, the following expression could be written:

(2) $$\left|{\tilde{U}}_{\text{ref}}\right|=C\;\left(1-\varepsilon_{\text{ref}}\right)\;\sigma \;\left|{\tilde{T}}_{\text{s}}^{\text{4}}\right|$$

where *T*_s_ (infrared source temperature) is the measured temperature (K) of the source, and *C* is a constant factor taking into account the emissivity of the source, the detector sensitivity, the voltage amplification, the shape factor and the optic transmission factor. $\text{σ }=\;5.67\;\times \;10^{-8}$ W·m^−2^·K^−4^ is the Stefan-Boltzmann constant.

Once the calibration was completed, the measurement of the unknown sample emissivity was achieved in the same experimental conditions and the hemispherical-directional reflectivity is given by:

(3) $$\rho^{\prime }=1-\varepsilon^{\prime }=K\frac{\left|{\tilde{U}}_{\text{m}}\right|}{\left|{\tilde{T}}_{\text{s}}^{\text{4}}\right|}$$

where $K=\frac{1}{C\text{σ}}$ is the constant obtained after calibration, $\varepsilon^{\prime }$ and $\left|{\tilde{U}}_{\text{m}}\right|$ are respectively the directional emissivity of the sample and the voltage amplitude (mV) measured by the detector.

In our case, some films seem to be semi-transparent materials and others are opaque materials. However, the experimental device was initially performed for opaque materials [17]. Thus, in these conditions, a modified procedure is required.

The indirect measurement method based on the second Kirchhoff law was applied, for the estimation of the directional spectral emissivity. This approach was used for the determination of the normal spectral emissivity for glasses from a room temperature up to 1200 K in a very wide range of frequencies [18]. For such types of materials, single crystals, and for moderate temperatures, the indirect method is very attractive because reflectivity and transmissivity measurements are currently performed.

When radiation strikes a surface area, the total energy in the incident electromagnetic waves (*I*_0_) is absorbed, reflected or transmitted. Therefore, the total energy can be expressed into three groups, characterized by three coefficients called absorptivity (α), reflectivity (ρ) and transmissivity (*t*) respectively. Relating to the second Kirchhoff law, the directional spectral absorptivity (α) of any radiator agrees with the directional spectral emissivity (ε), can be expressed as follows α = ε [19].

Therefore, the semi-transparent sample is placed under the cube with inner dimensions (10 × 10 × 10) cm^3^ and an opaque material is placed under the sample at a distance of 3 cm (Figure 2). Index 1 and 2 of parameters ρ and *t* are respectively attributed to the semi-transparent sample and opaque material.

The setup allows after calibration the estimation of the apparent reflectivity (ρ_ap_) of a semi-transparent sample. This parameter depends on the characteristics of the sample and also of the opaque material placed below.

ρ_ap_ can be expressed as follows:

(4) $$\begin{array}{l} {\text{ρ}}_{\text{ap}}\;=\;\frac{I_{\text{R}}}{I_{0}}\;=\;{\text{ρ}}_{1}\;+\;{\text{ρ}}_{2}\;t_{1}^{2}\;+\;{\text{ρ}}_{\text{1}}\;{\text{ρ}}_{\text{2}}^{\text{2}}\text{​}{\text{t}}_{\text{1}}^{\text{2}}\text{ + }{\text{ρ}}_{\text{1}}^{\text{2}}\;{\text{ρ}}_{\text{2}}^{\text{3}}\text{​}t_{\text{1}}^{\text{2}}\;+\;\ldots \;\\ \;=\;{\text{ρ}}_{\text{1}}\text{ + }{\text{ρ}}_{\text{2}}\;t_{\text{1}}^{\text{2}}\;\sum_{i=0}^{\infty }{\left({\text{ρ}}_{\text{1}}\;{\text{ρ}}_{\text{2}}\right)}^{i}={\text{ρ}}_{\text{1}}\;+\;\frac{{\text{ρ}}_{2}\;t_{1}^{2}}{1\;-\;{\text{ρ}}_{\text{1}}\;{\text{ρ}}_{\text{2}}} \end{array}$$

Several tests were performed for the calibration of the setup and also for the validation of the measurement method. For a ρ_2_ = 0 (black body placed under the semi-transparent sample), the measured reflectivity is ρ_1_ of the sample. For ρ_2_ = 1 (perfect mirror placed under the semi-transparent sample), the measured of the apparent reflectivity (ρ_ap_) is given by:

(5) $${\text{ρ}}_{\text{ap}}={\text{ρ}}_{\text{1}}\;+\;\frac{t_{1}^{2}}{1\;-\;{\text{ρ}}_{\text{1}}}$$

Indeed, the use of a black body and a fully reflective surface placed successively under the specimen allows the estimation of the semi-transparent sample properties. Thus, in this case the most important model parameters relating to the semi-transparent sample properties are ρ_1_ and *t*_1_. Besides, a sensitivity study of the model to these two parameters (ρ_1_, *t*_1_) was also performed and the results confirm that it is necessary to use both highly reflective and absorbent surfaces for an optimal estimation.

In this study, several materials with known properties (calibrate materials with known values of ρ_2_) were placed under the semi-transparent sample (LDPE/Al). These calibrated materials number six in total: smooth aluminium surface (ρ_2_ = 0.99); rough aluminium surface (ρ_2_ = 0.94); alumina surface (ρ_2_ = 0.26); carton surface (ρ_2_ = 0.12); black paint Nextel 811-21 (ρ_2_ = 0.03) and dimpled black foam (ρ_2_ = 0.01).

For each calibration material, the apparent reflectivity (ρ_ap_) was measured three times, allowing the calculation of an average value and a standard deviation for the three measurements.

The properties (ρ_1_ and *t*_1_) of the semi-transparent sample (LDPE/Al) are then estimated with knowledge of ρ_2_ by minimizing the squared deviation between the experimental data and the model described by Equation 4. Finally, relating to Rozenbaum *et al.* the emissivity ε_1_ is calculated according to [19]:

ε_1_ = 1 − ρ_1_ − *t*_1_ (6)

Flame retardancy was studied using a cone calorimeter (Fire Testing Technology). A horizontal sample sheet of squared section 100 × 100 mm^2^ was placed at 2.5 cm below a conic heater and insulated by rock wool. The samples were exposed to various heat fluxes (35, 50 and 75 kW/m^2^) in well-ventilated conditions (air rate 24 L/s) in the presence of a spark igniter to force the ignition. HRR was determined according to oxygen depletion (Huggett’s relation). The tests were performed according to the ISO 5660 standard [20]. Each formulation was tested twice.

**Figure 3:**
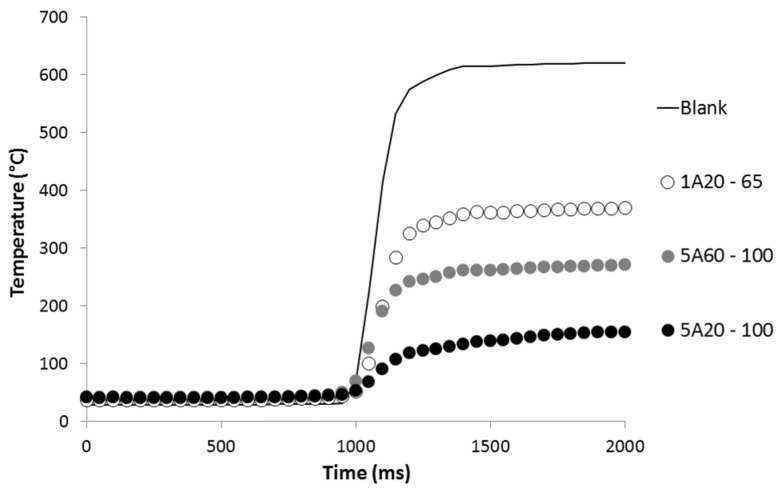
Temperature recorded by the infrared pyrometer during the epiradiator test for several films.

The thin films containing aluminum particles were also characterized by an “epiradiator test” instrumented with an infrared pyrometer (Optris, Berlin, Germany) as already described (see Figure 1 in [11]). Pure LDPE film was not tested using this device. 7 × 7 cm^2^ films are placed horizontally on a grid perforated in its center. The infrared pyrometer is placed perpendicularly to the surface below the specimen in order to measure the temperature through the grid hole. The temperature is recorded and corrected considering the true emissivity of the film measured as described above. After 1 s, films are exposed to a 500 W radiator (diameter 10 cm, made of opaque quartz) located 34 mm above the grid. In these conditions, the radiative incident heat flux from epiradiator on the upper surface is equal to 37 kW/m^2^. After a few seconds, the films retract and the test is stopped. Due to the semi-opaque behavior of the films, the temperature measured by the infrared pyrometer increases very quickly (see the temperature evolution for several films in Figure 3). The thermal radiation intercepted by the pyrometer comes both from radiation passing through the sample without being absorbed and from radiation emitted at the backside of the sample after absorption of the initial wave and transferred by conduction. Thus the heating rate value assesses the ability of the film to limit the heat transfer to the LDPE sheet in a cone calorimeter test.

Images of residues after cone calorimeter tests were obtained with a scanning electron microscope (SEM) (Quanta 200 SEM, FEI, Eindhoven, The Netherlands).

## 3. Results and Discussion

Some authors have focused on the dependence of the thermal radiative properties of a material on the heat source. Indeed the heat absorbance of a material depends on the spectral distribution of the radiation, which depends itself on the heat source and its temperature. Linteris *et al.* have advised measuring the thermal radiative properties on the wide wavelength range [21]. Conical resistance as in a cone calorimeter was found to behave as a black body [22,23]. Boulet *et al.* have shown that the peak of emission of the cone heater part is centred between 1500 and 2500 cm^−1^ depending on the heat flux (in the range 10–60 kW/m^2^), *i.e.*, few micrometers [23]. This peak is included in the spectral range investigated by our device to measure the thermal radiative properties of our materials. Then, the values measured are meaningful when investigating the dependence of ignition on the thermal radiative properties.

### 3.1. Thermal-Radiative Properties of the Coating Films

The results of emissivity, reflectivity and transmissivity measurements are presented in Table 3. The procedure of semi-transparent materials (opaque material under sample) was applied for all samples except 5A20 (seems to be opaque).

**Table 3:** Emissivity, reflectivity and transmissivity coefficients of coatings.

Coatings	ρ_1_	*t*_1_	ε_1_
1A20-65	0.231 ± 0.005	0.260 ± 0.012	0.509 ± 0.013
1A20-100	0.245 ± 0.006	0.192 ± 0.012	0.563 ± 0.013
3A20-100	0.349 ± 0.001	0.110 ± 0.003	0.541 ± 0.004
5A20-100	0.369 ± 0.006	0	0.631 ± 0.006
5A40-100	0.372 ± 0.009	0.132 ± 0.040	0.497 ± 0.041
5A60-100	0.370 ± 0.002	0.156 ± 0.006	0.475 ± 0.007

**Figure 4:**
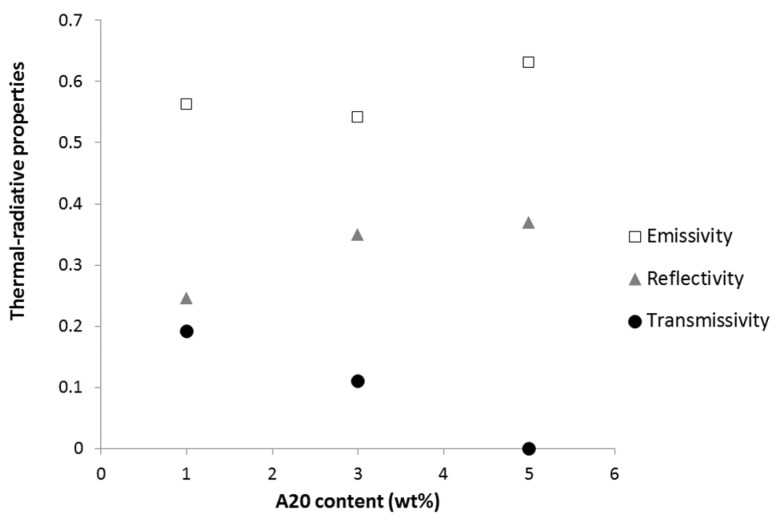
Effect of A20 content on emissivity, reflectivity and transmissivity of coatings (error bars are lower than the data points size).

Figure 4 shows the variation of emissivity, reflectivity and transmissivity coefficients of A20 films as a function of aluminum content. We notice an increase of reflectivity when the aluminum concentration increases. The transmissivity tends to decreases substantially when the particles concentration increases until we obtain an opaque sample corresponding to 5% of A20 grade (*i.e.*, 3.5 wt % of aluminum). The evolution of the emissivity is less distinct; the sample 5A20 (3.5 wt % of Al) seems to exhibit a higher emissivity than 1A20 (0.7 wt % of Al) and 3A20 (2.1 wt % of Al) ones. In fact the sample 5A20 is an opaque material and in this case, the emissivity was calculated as ε = 1 − ρ. In fact, some polymers are highly transparent materials in the infrared range, and the emissivity of such composites strongly depends on the properties of the metallic particles themselves and the contact or porosity between particles [13].

Yu *et al.* have also observed a decrease of emissivity when increasing copper particle content in EPDM [13]. Chen Hu *et al.* have also shown that the emissivity of polysiloxane/aluminum opaque composites decreases with increasing aluminum content, at least up to 30 wt % of aluminum [14]. In both cases, the metallic particle content is much higher than in our work and it may not be fully comparable. Just for example, if we accept to add the emissivity and transmissivity values as for the opaque materials, for the sample 1A20 and 3A20, we obtain also a decrease of the estimated value for all samples (1A20, 3A20 and 5A20) with the increasing of aluminum content.

**Figure 5:**
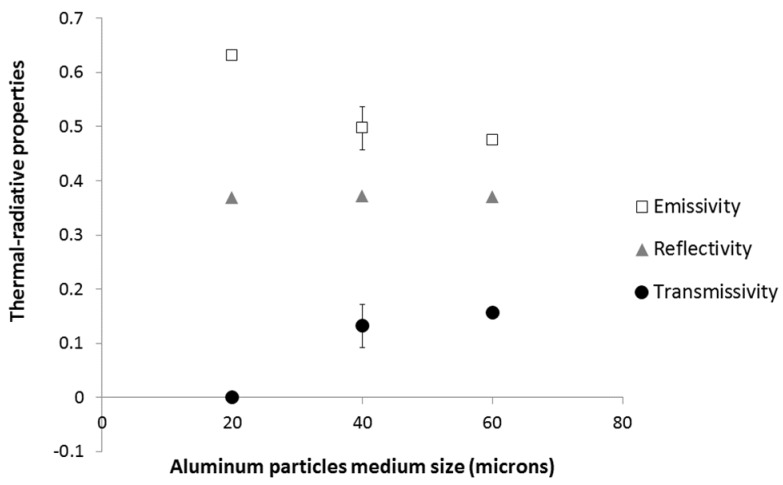
Effect of aluminum particles medium size on emissivity, reflectivity and transmissivity of 5A20, 5A40 and 5A60 coatings.

The effect of aluminum particles of medium size on emissivity, reflectivity and transmissivity is presented in Figure 5. It is noted that the size of the aluminum particles has no effect on the reflectivity, but it significantly affects both emissivity and transmissivity properties. The increase of particle size at a constant weight concentration (3.7 wt % of Al) tends to increase the transmissivity, and to reduce the emissivity.

Finally, a characterization of two films (1A20) with the same filler content and different thicknesses (65 and 100 μm) was performed. The results highlight that an increase in the thickness of the film tends to increase the emissivity and the reflectivity and reduce the transmissivity. This effect was also observed by Chen Hu *et al.* for polysiloxane filled with aluminum particles [14].

### 3.2. Cone Calorimeter Results

All formulations exhibit the same fire behavior at 35 kW/m^2^. The peak of heat release rate (pHRR) is around 700 kW/m^2^. The LDPE matrix is fully decomposed (residue yield is negligible) and the effective heat of combustion is close to 32 kJ/g. This last value is lower than the effective heat of complete combustion for polyethylene (40–42 kJ/g as measured in a pyrolysis-combustion flow calorimeter) leading to a combustion efficiency in the range 0.75–0.8.

**Figure 6:**
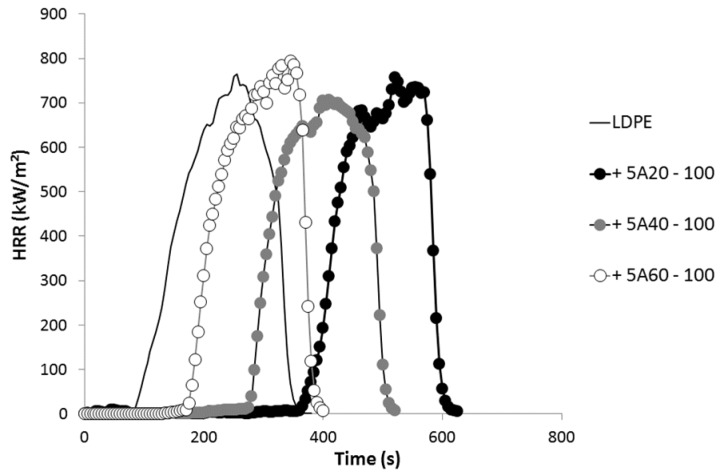
Heat release rate curves obtained in a cone calorimeter test at 35 kW/m^2^ for various formulations.

Only time-to-ignition (TTI) is significantly modified by the different coatings at the top surface of the samples. TTI increases from 76 s for pure LDPE to 397 s for LDPE coated with 5A20-100 coating. Therefore, all the heat release rate curves are similar but shifted to different TTI according to the coating (see Figure 6). As already reported by Schartel *et al.* [15], the coatings act as infrared mirrors delaying ignition, but do not change the behavior of the material once ignited. The maximum increase in TTI obtained in our study is in the same range (but slightly lower) than the increases observed by Schartel *et al.* [15]. In their study involving a three-layer coating, TTI increases from 58 to 537 s for PA66 and from 82 to 459 s for PC.

**Figure 7:**
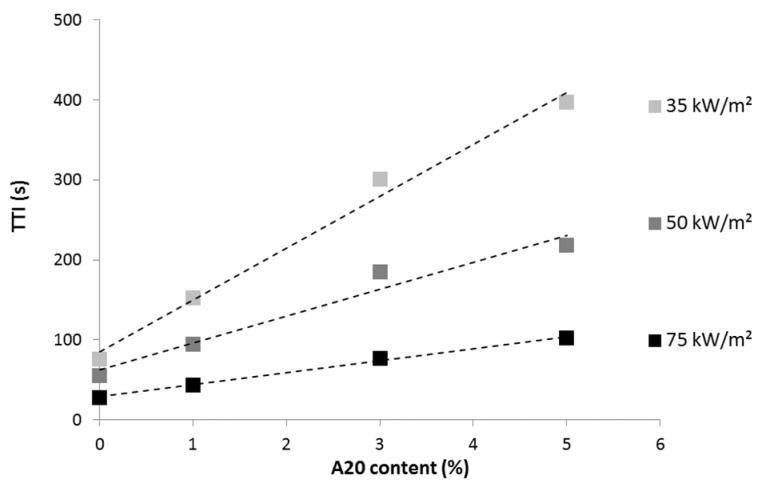
Time-to-ignition (s) *versus* A20 content (thickness 100 μm) at various heat fluxes.

Considering 100 μm-thick films containing A20 particles, it is noteworthy that the effect of the coating is more significant at a low heat flux (Figure 7). Indeed, the ratio between the TTI of LDPE coated with 5A20 containing film and TTI of pure LDPE is 5.2, 3.9 and 3.7 at 35, 50 and 75 kW/m^2^, respectively. It must also be noted that at each heat flux, the TTI increases linearly when increasing aluminum particle content. The TTIs obtained with the 65 μm-thick film containing A20 particles are slightly lower than those measured with the 100 μm-thick film containing the same amount of A20 particles. For example, at 50 kW/m^2^, the TTI is 90 s *versus* 94 s for the thicker film.

**Figure 8:**
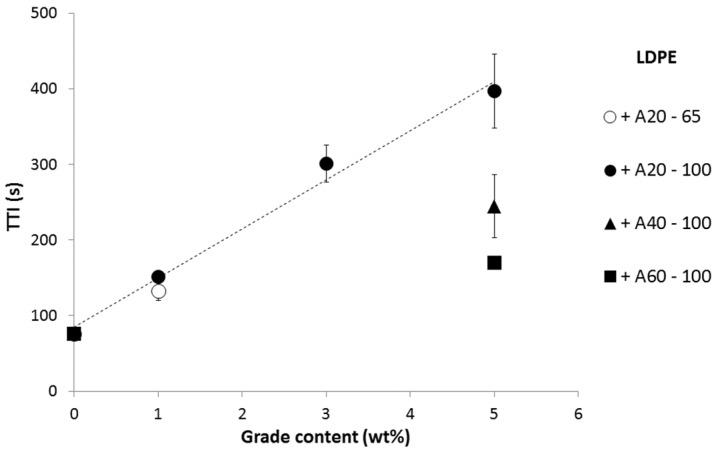
Time-to-ignition (s) *versus* grade content in the coating at 35 kW/m^2^.

Figure 8 summarizes the TTI obtained at 35 kW/m^2^ for various formulations. Besides the aluminum particle content, it appears that TTI also depends on the grade used. A20 is much more efficient than A40, and A60 is the least efficient grade. The thickness of the coating is another parameter affecting the time-to-ignition. LDPE coated with 65 and 100 μm-thick 1A20 coatings exhibits a TTI of 133 and 152 s, respectively, at 35 kW/m^2^. This influence appears quite limited and further study is needed to confirm this preliminary result.

**Figure 9:**
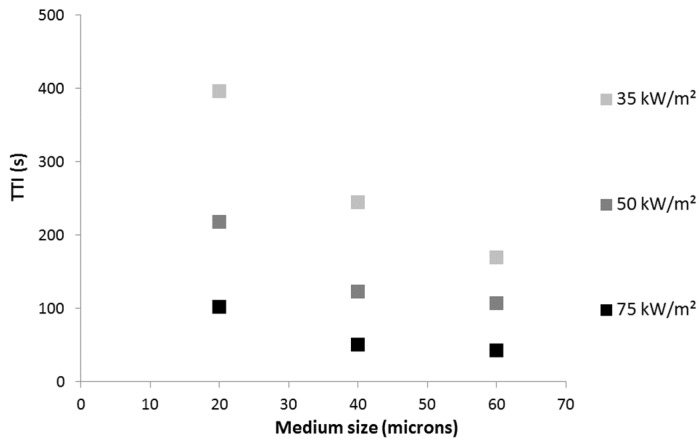
Time-to-ignition (s) *versus* medium particle size for LDPE coated with 5A20, 5A40 and 5A60 coatings at various heat flux.

The efficiency of the various aluminum particles to delay ignition depends on their shape and size. Particles from A40 and A60 have the same shape (corn flakes) but A40 particles are smaller (medium size is around 40 μm *versus* 60 μm for A60). Despite its higher absorptivity, 5A20 coating exhibits a much higher efficiency, which could be related to the smallest particle size (20 μm) or alternatively to their specific shape and to their leafing nature (Figure 9). Leafing pigments may tend to float to the surface of the coating and to align parallel to the surface of the coating. The relative influence of these parameters (size, shape and leafing nature) is out of the scope of this article and needs further study. Our primary objective is to relate the efficiency of various grades to their thermal-radiative properties.

**Figure 10:**
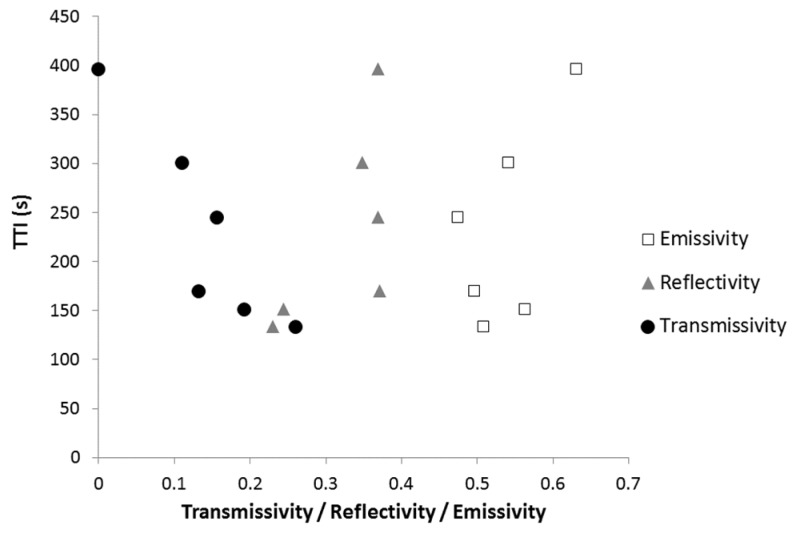
Time-to-gnition (s) at 35 kW/m^2^ *versus* thermal-radiative properties of the coatings.

Figure 10 plots the TTI measured in a cone calorimeter test at 35 kW/m^2^ *versus* the thermal-radiative properties of the coatings: reflectivity, transmissivity and emissivity. There is no simple relationship between TTI and one of these properties. This means that the ignition depends on a complex combination of these properties.

**Figure 11:**
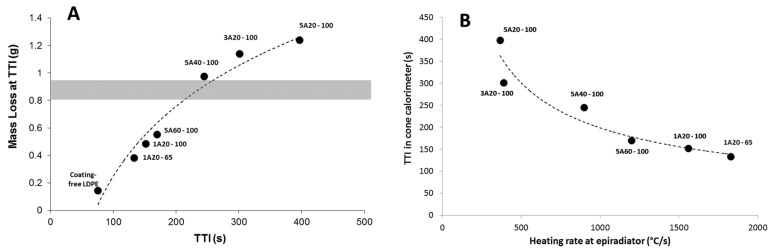
(**A**) Masse loss at time-to-ignition (TTI) for all formulations at 35 kW/m^2^ (the grey zone corresponds to the mass of low-density polyethylene (LDPE) in the coating); (**B**) Time to ignition in cone calorimeter at 35 kW/m^2^ *versus* heating rate measured using epiradiator (the dotted line is only a guideline for the eyes—labels correspond to the coating thermocompressed on the top surface of LDPE sheet).

Figure 11 provides some evidences that TTI is not only dependent on the coating emissivity. Figure 11A shows that the mass loss at ignition exceeds 1 g for some formulations. The coating corresponds to around 0.8–0.9 g of the total mass of the sample. This means that the ignition occurs when LDPE in the sheet (and not only in the coating) starts degrading and contributes to the fuel release. Then, the heating of the sheet is of primary importance. This heating depends not only on the reflectivity but also on the transmissivity of the coating.

Similarly, Figure 11B plots the TTI measured in cone calorimeter at 35 kW/m^2^ with the heating rate at the bottom surface of the coating as measured using an epiradiator. Keep in mind that this heating rate depends on many properties of the coating: thermal-radiative properties and also thermal conductivity. A good correlation can be found between the heating rate and the time-to-ignition, showing once again that ignition depends on several coating properties.

Two equations have been proposed to predict the time-to-ignition *versus* the applied heat flux [2]. In these equations, emissivity is not present explicitly. Actually, net heat flux must be considered rather than external heat flux. Net heat flux is equal to the product of external heat flux and emissivity. The modified equations (*i.e.*, when emissivity is taken into account explicitly) are given below. The first one corresponds to thermally thick materials and the second one to thermally thin materials:

(7) $$\text{TTI}=\;\frac{\text{π}}{4}k\text{ρ}c{\left[\frac{T_{\text{ig}}-T_{0}}{\text{ε}{\dot{q}}_{\text{ext}}-\text{CHF}}\right]}^{2}$$

(8) $$\text{TTI}=\;l\text{ρ}c\frac{T_{\text{ig}}-T_{0}}{\text{ε}{\dot{q}}_{\text{ext}}-\text{CHF}}$$

with *k* the thermal conductivity, ρ the density, *c* the specific heat, *T*_ig_ the surface temperature at ignition, *T*_0_ the room temperature (25 °C), ε the emissivity, ${\dot{q}}_{\text{ext}}$ the applied heat flux and CHF the critical heat flux.

The dependence of TTI on heat flux is different according to the thermal behavior of the material. Then, a method to assess if the material is thermally thin or thick is to draw ($\sqrt{\text{TTI}}$)^−1^ or TTI^−1^ *versus* the heat flux. If the curve ($\sqrt{\text{TTI}}$)^−1^ *versus* heat flux is linear, the material is thermally thick. If the curve TTI^−1^ *versus* heat flux is linear, the material is thermally thin. Unfortunately, for all our materials, both curves seems to be linear. In that case, this method is of poor practical interest. To determine the thermal behavior of our materials, we need to know all their properties to calculate TTI using Equations (7) and (8).

**Figure 12:**
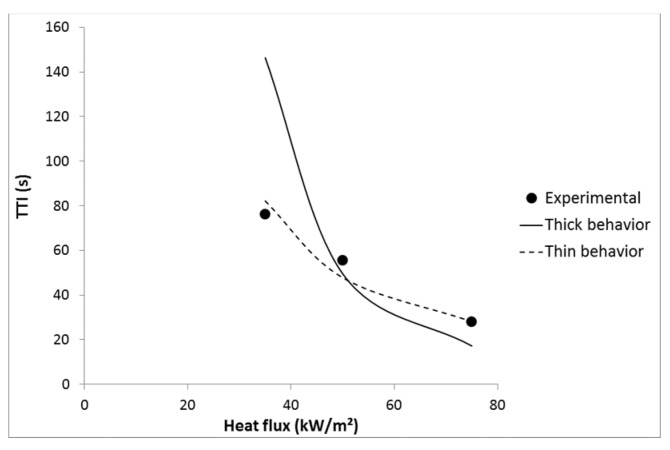
Experimental and predicted TTI *versus* heat flux for LDPE.

We assume that the properties of LDPE are the same as reported by Lyon’s report [1]: emissivity 0.92, density 925 kg/m^3^, thermal conductivity 0.38 W·m^−1^·K^−1^, specific heat 1.55 kJ·kg^−1^·K^−1^, temperature at ignition 377 °C and CHF 13 kW/m^2^. Using these values, time-to-ignition can be well predicted using Equation (8) corresponding to a thermally thin material (see Figure 12). Indeed, pure LDPE does not strongly absorb in the infrared region. Linteris *et al.* have measured the absorption coefficient of 11 thermoplastics and found that HDPE, PP and PS exhibit the lowest absorption coefficient. In the case of HDPE, approximately only 50% of the incident heat is absorbed in the first 500 μm [21]. Therefore its behavior is thermally thin even at high heat flux.

It must be noted that Equations (7) and (8) do not take into account some phenomena. For example, thermophysical and thermal radiative properties are measured at ambient temperature. Their evolution at high temperature and when the material starts degrading is not known. Moreover, the absorption coefficient changes with the thickness as shown by Linteris *et al.* [21] even if this change is small for polyethylene. Finally, the absorption depends on the spectral distribution of the radiation which changes with the temperature of the heat source [16,21,22,24]. As a result, the absorptivity of the materials should change when heat flux changes. Despite these limitations, Equation (8) fits the experimental TTI of pure LDPE well.

For coated LDPE, all the properties listed above are maintained except the emissivity. Indeed, the presence of a very low aluminum amount into only a thin layer at the top of the surface can not seriously affect these properties. According to Rozenbaum *et al.* the emissivity ε_1_ can be expressed by Equation (6) for semi-transparent materials such as our coatings (transmissivity is not null) [19]. Nevertheless, when these coatings are thermo-compressed onto an LDPE sheet, we assume that the materials are opaque and then emissivity should expressed by Equation 3 (transmissivity is null) [19]. This is the reason why emissivity of the coated sheets (LDPE sheet + coating) is calculated by adding the emissivity and the transmissivity values of the corresponding coating alone.

**Figure 13:**
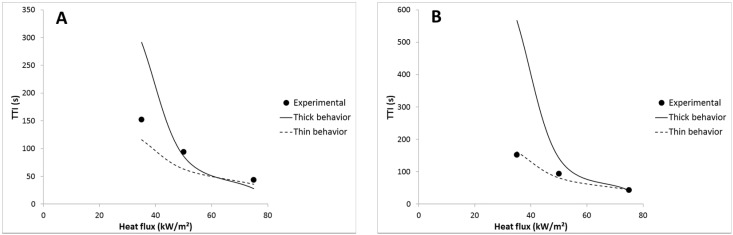
Experimental and predicted TTI *versus* heat flux for LDPE coated with 1A20-100 film: (**A**) emissivity = 0.76, (**B**) emissivity = 0.65.

For most of the formulations (with 1A20, 5A40 and 5A60 coatings), the time-to-ignition can be predicted well considering a thermally thin behavior as for LDPE, but emissivity must be adjusted to a lower value than expected (example for LDPE coated with 1A20-100 coating in Figure 13). In other words, the emissivity of the coated sheets calculated as the sum of the emissivity and the transmissivity of the coating is too high to match the experimental TTI using Equation (8). The emissivity used to fit properly the experimental TTI is called “fitted emissivity”. Its value is chosen to match at best the experimental TTI measured at the three heat fluxes (35, 50 and 75 kW/m^2^).

To explain why a lower emissivity than expected must be considered, it must be noted that the decrease in emissivity is due to inert aluminum particles. While LDPE degradation occurs, the aluminum concentration in the coating increases. Then the emissivity may continuously decrease.

**Figure 14:**
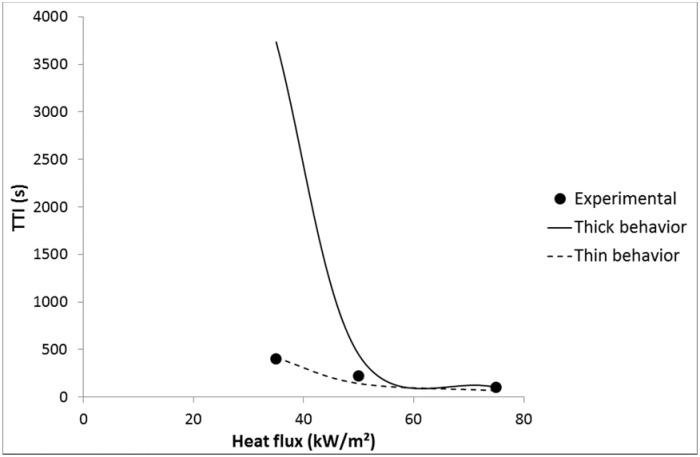
Experimental and predicted TTI *versus* heat flux for LDPE coated with 5A20-100 coating.

Nevertheless, for LDPE coated with 3A20 and 5A20 coatings, choosing a lower emissivity is not enough to fit well the experimental time-to-ignitions with a thermally thin behavior. For example, an emissivity of 0.48 allows matching the TTI at 35 kW/m^2^ for LDPE coated with 5A20 coating, but the TTI at 75 kW/m^2^ is then underestimated (69 s *versus* 103 s). On the contrary, considering a thermally thick behavior at 75 kW/m^2^ allows matching the experimental TTI (see Figure 14). In other words, it is necessary to choose a lower emissivity and to consider a change of the thermal behavior from thin to thick when heat flux increases from 35 to 75 kW/m^2^. At intermediate heat flux, the behavior appears hybrid. A thermally thick behavior at high heat flux allows a high time-to-ignition to be maintained. This change appears coherent with the thermal-radiative properties of the coating films. Indeed, 3A20 and 5A20 coatings exhibit the lowest transmissivity among all coatings. 5A20 coating is even the only fully opaque coating.

Table 4 summarizes the estimated and the fitted emissivities of the coated sheets. Recall that the estimated emissivity was calculated by adding the emissivity and the transmissivity values of the coating (measured in Table 3). The difference between both values is the highest for the sheets coated with 3A20 and 5A20 films. While the corresponding coatings exhibit the lowest transmissivity, it can be assumed that a low transmissivity promotes a relatively fast degradation of LDPE in the coating and then a fast increase in aluminum particle concentration, leading to a low emissivity during burning.

**Table 4:** Estimated emissivity and fitted emissivity of coated sheets

Sheet	Estimated Emissivity 1 − ρ_1_	Fitted Emissivity ε_2_	(1 − ρ_1_) − ε_2_
LDPE	-	0.92 [25]	-
1A20-65	0.77	0.7	0.07
1A20-100	0.76	0.65	0.11
3A20-100	0.65	0.53	0.12
5A20-100	0.63	0.48	0.15
5A40-100	0.63	0.56	0.07
5A60-100	0.63	0.64	−0.01

**Figure 15:**
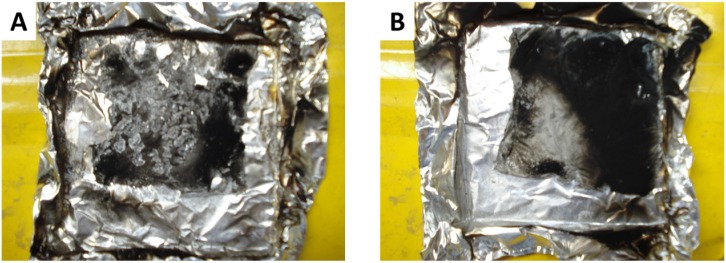
Residues at the end of the cone calorimeter test. (**A**) LDPE coated with 5A40 coating. (**B**) LDPE coated with 5A20 coating.

The highest efficiency of 3A20 and 5A20 coatings can be also related to the ability of the A20 particles to form a thin but cohesive aluminum film during the coating degradation. Figure 15 shows the residue of LDPE coated with 5A20 or 5A40 films after a cone calorimeter test at 35 kW/m^2^. The residue from 5A20 is a very thin but cohesive aluminum film which can be removed without decomposing. It retracts during the test but covers the major part of the surface. Residue from LDPE coated with 3A20 film exhibits a similar aspect. The residues from LDPE coated with 5A40 films and all other films are in pieces and do not cover a significant fraction of the surface.

**Figure 16:**
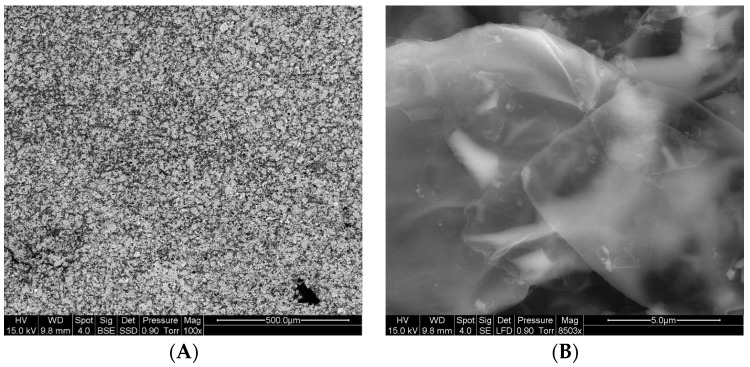
Scanning electron microscope (SEM) observations of 5A20 film residue at two different scales. (**A**) Low magnification; (**B**) High magnification.

SEM observations of the residue from LDPE coated with 5A20 coating are shown in Figure 16. It seems that particles overlap and are well-aligned parallel to the surface (probably due to the leafing effect). It can be suggested that this alignment favours a low transmissivity and facilitates the formation of a thin and cohesive aluminum film.

## 4. Conclusions

Partially or fully opaque LDPE coating films with controlled emissivity were prepared by incorporating a small amount of various micron-sized aluminum particles. These coatings were able to delay the ignition of pure LDPE 4 mm-thick sheets from 80 to 400 s at a heat flux of 35 kW/m^2^. This effect is more prominent at low heat flux.

The relation between the thermal-radiative properties of the films and their efficiency in delaying ignition was elucidated. A lower emissivity reduces the net heat flux at the surface of the sample. As the coating degrades, the aluminum concentration increases, leading to decreasing emissivity. A low transmissivity film exacerbates this effect. Moreover, if the films exhibit a low transmissivity, this changes the thermal behavior from thin to thick at high heat flux. Such a change allows a high time-to-ignition to be maintained.

Finally, it appears that the most efficient coatings are obtained with the smallest aluminum particles which also exhibit a leafing behavior. This leafing effect seems to be related to the cohesion of the aluminum film which is formed during the degradation.

The strategy proposed is simple and allows a high TTI to be reached. Moreover, it is versatile (similar results were obtained with other coated polymers—data not shown). Nevertheless, such an infrared-mirror effect should only be available using a radiative source. When the sample is heated by a contacting flame, the effect may vanish.
